# Camouflage Modalities of Treatment for Skeletal Class III Malocclusion in Adults—A Narrative Review

**DOI:** 10.3390/jcm15103680

**Published:** 2026-05-11

**Authors:** Valentina Rutili

**Affiliations:** Private Practice in Orthodontics, 50100 Florence, Italy; valentina.rutili@libero.it

**Keywords:** adult, camouflage, orthodontics, malocclusion, angle class III

## Abstract

**Background**: Orthodontic camouflage in Class III patients is a widely used treatment approach. However, its application is subject to specific clinical indications. This narrative review aims to synthesize the current scientific evidence and provide an overview of the available methods, their advantages and limitations, in order to guide the appropriate management of Class III camouflage cases. **Methods**: A literature search was carried out using five main scientific databases without restrictions. Inclusion criteria were all types of articles published on various orthodontic camouflage techniques for Class III malocclusion in adult patients. The string searches included “camouflage” and “Class III malocclusion”. In addition, a manual search was performed to identify further relevant articles. The methodological quality was assessed using the Oxford Centre for Evidence-Based Medicine (OCEBM) classification. **Results**: This narrative review synthesizes 128 studies on orthodontic camouflage in adult Class III malocclusion. Among the 128 articles included, 110 (86%) were case reports or small case series, corresponding to level 4–5 evidence. The remaining studies were observational in design, most of them retrospective, corresponding to level 2–3 evidence. Extractive or non-extractive treatment can be used for non-surgical treatment of a Class III in adults. In recent years, aesthetic techniques, such as clear aligners or lingual fixed appliances, have been efficiently performed. Carriere Motion III is a fast and efficient method to mask a Class III occlusal relationship. Moreover, the use of temporary anchored devices (TADs) has proven to be an effective and minimally invasive method for managing mandibular distalization and a retraction of the lower incisors. **Conclusions:** Although most reports are case-based, recent advances such as TADs and clear aligners offer effective non-surgical alternatives for selected mild-to-moderate cases. Careful patient selection remains critical. The evidence was low-quality, and further prospective studies are needed.

## 1. Introduction

Skeletal Class III malocclusion is characterized by an anteroposterior discrepancy between the maxilla and the mandible, which may result from mandibular prognathism, maxillary retrusion, or a combination of both [1]. This skeletal imbalance leads to an altered interarch relationship, often associated with functional limitations, aesthetic concerns, and a negative impact on the patient’s quality of life [2,3].

In adults, treatment options for Class III malocclusion primarily include orthognathic surgery and dentoalveolar camouflage. Although orthognathic surgery is considered the gold standard for correcting severe skeletal discrepancies [4,5], it is not always feasible or accepted by patients. Reluctance toward surgery may arise from concerns about potential complications [6], satisfaction with the existing facial profile [7], financial or psychological factors [8,9], or the presence of systemic comorbidities that contraindicate general anesthesia [10]. Moreover, surgical correction presents inherent biological limitations and a potential risk of relapse [11,12].

In such circumstances, orthodontic camouflage represents a valid alternative for non-growing patients with mild-to-moderate Class III malocclusion or for those unwilling or unable to undergo surgery [9,13,14]. The term camouflage refers to a dentoalveolar approach aimed at improving occlusal function and facial harmony through controlled tooth movement, without addressing the underlying skeletal discrepancy [7,14,15]. A variety of techniques have been proposed to achieve this goal, including the Carrière Motion III appliance and Multiloop Edgewise Archwire (MEAW) technique, each requiring precise case selection to ensure optimal outcomes.

Previous systematic reviews have examined camouflage therapy for Class III malocclusion [16]; however, recent techniques (such as clear aligner mechanics and skeletal anchorage systems) have not been comprehensively evaluated. Therefore, this narrative review aims to synthesize the current evidence on camouflage treatment for adult Class III malocclusion, with a focus on patient selection criteria, treatment modalities, and clinical outcomes. Specifically, it aims to address the following question: which orthodontic techniques offer the most effective dentoalveolar compensation for adult Class III patients with specific skeletal and dental characteristics?

## 2. Materials and Methods

This article was written in accordance with SANRA principles for conducting a narrative review [17].

**Figure 1:**
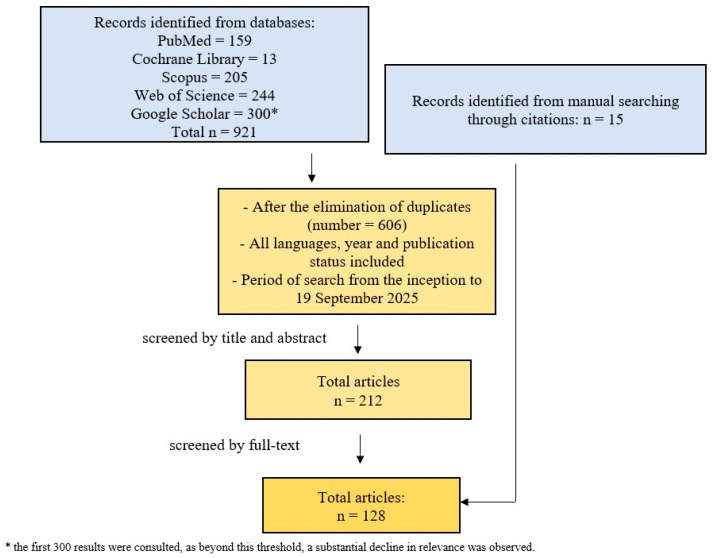
Flowchart of the research conducted.

A non-systematic literature search was performed across five databases, i.e., PubMed, Scopus, Web of Science, Cochrane Library and Google Scholar, from their inception to 19 September 2025. No language, publication, or year restrictions were applied. Figure 1 illustrates the framework of the research and selection process for articles according to a simplified PRISMA flow diagram [18]. Search words through databases included the terms “camouflage” and “Class III malocclusion”. Articles were included if they reported on camouflage orthodontic treatment in adult Class III patients. Both extraction and non-extraction cases were considered. All types of studies were included to capture the full range of therapeutic approaches. Studies focusing on surgical treatment or growing patients were excluded.

Formal risk-of-bias assessment tools were not applied, as this review aimed to provide a qualitative overview rather than a quantitative synthesis. However, methodological rigor and sample representativeness were considered during data interpretation.

The methodological quality of the included studies was qualitatively assessed according to the Oxford Centre for Evidence-Based Medicine (OCEBM) 2011 Levels of Evidence framework [19].

## 3. Results

After the removal of 306 duplicate records, 315 unique articles were identified and screened. Title and abstract screening led to the exclusion of 103 studies that did not address Class III camouflage treatment or were unrelated to the research focus. The remaining 212 articles underwent full-text evaluation for eligibility.

Following the full-text assessment, 128 studies met the inclusion criteria and were retained for qualitative synthesis (Figure 1). Among these, 110 were case reports or small case series describing both extraction and non-extraction treatment modalities. Observational studies accounted for a smaller proportion of the included literature (18 articles), with two of them reporting data from the same patient sample [20,21].

Broad inclusion criteria were intentionally applied to encompass a comprehensive range of treatment approaches, including historically relevant publications that contribute to the understanding of Class III camouflage therapy. The inclusion of older studies was justified by the need to illustrate the evolution of clinical concepts and biomechanical strategies over time.

A summary of the observational studies included is presented in Table 1.

The findings were grouped thematically according to treatment modality (e.g., fixed appliances, aligners, or skeletal anchorage) and clinical indication (mild, moderate, or borderline cases). Prominence was placed on identifying common diagnostic criteria and biomechanical strategies.

### 3.1. Quality Appraisal of the Included Evidence

Among the 128 studies included, 86% were case reports or small case series (level 4–5 evidence, according to the Oxford CEBM classification [19]), 13% were retrospective observational studies (level 3), and only 1% provided prospective data (level 2). Therefore, the overall strength of evidence supporting camouflage approaches in adult Class III cases can be considered low. Most studies lacked control groups, standardized outcome measures, or long-term follow-up.

Table 2 provides a qualitative appraisal of the evidence level assigned to each treatment category.

### 3.2. Selection Criteria for a Class III Camouflage

In patients with skeletal Class III malocclusion, several diagnostic and anatomical factors must be carefully evaluated before initiating camouflage treatment. These include the severity of the skeletal discrepancy, the patient’s chief complaint and self-perception, the vertical facial dimension, and the morphology of the dentoalveolar bone. Table 3 summarizes the main clinical considerations related to case selection. The decision between orthodontic camouflage and orthognathic surgery depends largely on the patient’s primary concern and self-image [38].

In borderline Class III cases, discriminant cephalometric parameters such as the Holdaway angle and Wits appraisal are useful in determining whether camouflage treatment is feasible or if surgical correction is indicated [39]. Dento-alveolar limitations also play a crucial role. The inclination of the incisors must permit adequate dental compensation (specifically, maxillary incisor proclination and mandibular incisor retroclination) without exceeding the biological limits of the alveolar bone [40]. When further compensation risks inducing alveolar dehiscence, fenestration, or periodontal compromise, camouflage treatment should be avoided [23,41]. Similarly, adequate alveolar bone thickness and mandibular symphysis morphology are essential to allow controlled tooth movement during retraction or uprighting [13,14].

Facial esthetics is another critical determinant. Patients with a markedly concave or unaesthetic profile are unlikely to benefit from camouflage, as this treatment may further accentuate facial imbalance. Conversely, individuals with mild skeletal discrepancies and relatively acceptable facial harmony represent better candidates [4,13].

Finally, the magnitude of the skeletal discrepancy must remain within the limits of dentoalveolar compensation. When the anteroposterior discrepancy is severe, camouflage becomes biologically and esthetically inappropriate, even if other diagnostic criteria are favorable [42,43]. Likewise, patients with excessive vertical divergence generally respond poorly to the extrusive mechanics required for camouflage; therefore, low- or normo-angle patterns are more suitable [9,25].

### 3.3. Treatment Approaches for Class III Camouflage

**Figure 2:**
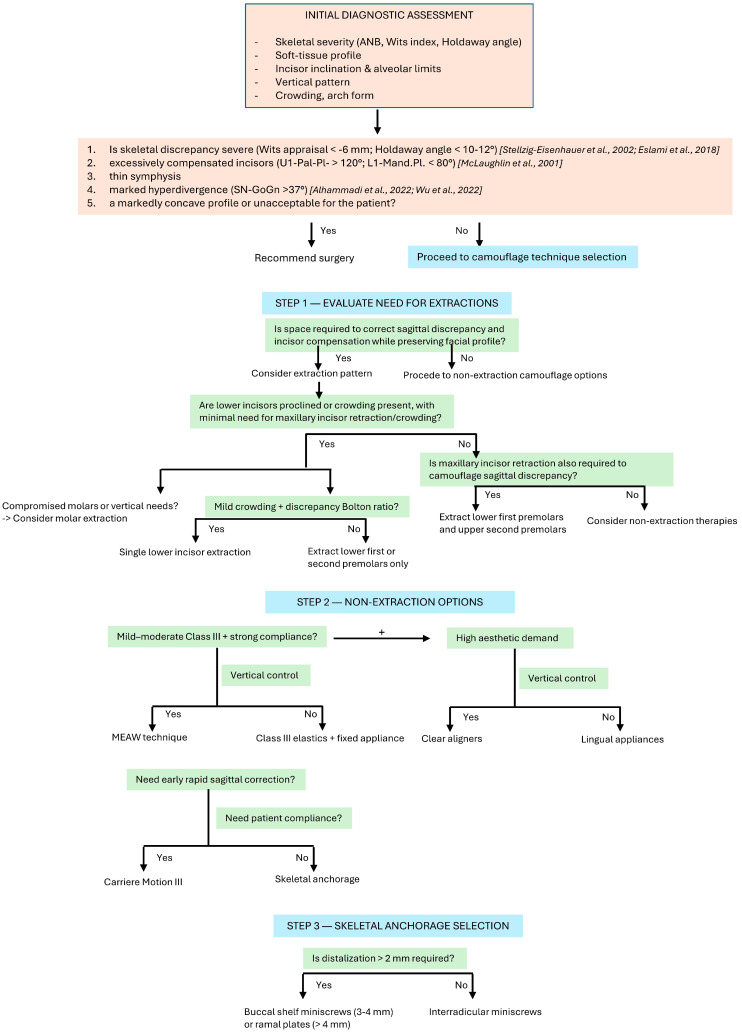
Decision flowchart for technique selection in adult Class III camouflage treatment [4,9,25,39,40].

A variety of orthodontic strategies have been proposed for the camouflage management of Class III malocclusion. Depending on the severity of the skeletal and dental discrepancy, as well as the amount of crowding, treatment may involve extraction or non-extraction protocols [4,44]. Figure 2 shows a decision flowchart for technique selection in adult Class III camouflage treatment.

The traditional extraction-based approach generally involves the removal of two mandibular premolars to allow dental compensation, although extraction of a single lower incisor may be considered in less severe cases.

In addition to conventional extraction mechanics, several non-surgical protocols have been described to address Class III relationships, including the Carrière Motion III appliance, Multiloop Edgewise Archwire (MEAW) technique, and distalization strategies using temporary anchorage devices (TADs). These modalities differ in their biomechanical principles but share the common objective of achieving a stable, functional occlusion while improving facial esthetics.

For clarity, the studies included in this review were categorized according to two main criteria: (1) the type of treatment protocol (extraction vs. non-extraction) and (2) the type of orthodontic appliance used (labial fixed appliances, self-ligating systems, lingual appliances, clear aligners, Carrière Motion III, MEAW, or skeletal anchorage-assisted techniques).

Table 4 presents a summary of the characteristics of camouflage techniques for adult Class III malocclusion.

### 3.4. Extraction-Based Camouflage Approaches

The decision to perform extractions in Class III camouflage depends primarily on the degree of crowding, the curve of Spee, and the severity of the skeletal discrepancy. When mandibular incisor retroclination is required to achieve a positive overjet, extraction of the first (or the second) premolars may be indicated. However, studies report variable outcomes in terms of the amount of incisor retroclination, likely due to differences in initial crowding, curve of Spee depth, and pretreatment morphology [45]. In such cases, most of the extraction space is used for alignment and leveling, with limited space remaining for retraction. Physiological mesial drift of the posterior teeth may also contribute to space closure [41]. In patients with mild crowding and a favorable Bolton tooth-size ratio, extraction of a single mandibular incisor can be considered as an alternative option [46].

Extraction treatment, particularly when combined with arch coordination and elimination of deleterious oral habits, disrupts the pre-existing occlusal equilibrium and allows the establishment of a new functional balance within the stomatognathic system [41].

Patients presenting with high-angle growth patterns, open-bite tendencies, and crowding may particularly benefit from extraction-based camouflage approaches [47].

#### 3.4.1. Extraction of Premolars

Extraction of premolars remains a common strategy for correcting the anteroposterior discrepancy in Class III camouflage cases. The most frequent protocol involves removing the upper second and lower first premolars, typically indicated in patients with anterior crossbite, mesial molar relationship, moderate upper crowding, proclined lower incisors, and mild skeletal deformity [48].

When both arches are protrusive with significant upper crowding, extraction of all first premolars may be indicated to facilitate mandibular antero-rotation and improve facial balance [49]. Exclusive mandibular premolar extraction is another widely used approach, suitable for patients with concave profiles in whom upper extraction could worsen facial retrusion [50]. However, this option should be reserved for cases requiring substantial retraction of the lower incisors [20]. In a retrospective study, no differences were found between extraction of the lower premolars and distalization with Class III elastics or TADs regarding treatment time, treatment efficiency and occlusal aspects [31].

Careful control of tooth movement is essential, as long-term follow-up studies have shown adaptive remodeling of the anterior mandibular alveolar bone [21].

In patients exhibiting transverse compensation, premolar extraction can be combined with skeletal or surgically assisted maxillary expansion when upper teeth are buccally inclined and lower teeth lingually inclined [51].

Conversely, extraction of four premolars in severe Class III cases should be approached with caution, as it may increase facial concavity due to upper lip retrusion [52].

#### 3.4.2. Extraction of a Lower Incisor

Extraction of a single mandibular incisor may be appropriate in cases with mild anterior crossbite, good posterior intercuspation, and an acceptable facial profile [46]. This approach offers several advantages, including reduced treatment time, minimal alteration of intercanine width, and improved long-term stability [46,53]. A systematic review indicated that outcomes following lower incisor extraction can be stable and esthetically satisfactory when case selection is appropriate [46].

Nonetheless, several limitations must be considered in adult patients: potential development of black triangles due to papillary loss, tooth-size discrepancies affecting occlusal relationships, and concerns about visible extraction spaces [54].

Reduced contact points may weaken occlusal balance, requiring long-term monitoring [55]. Furthermore, a small Bolton discrepancy may result in increased overjet [56,57].

Moreover, relapse of the alignment should be carefully contained, due to the reduced, even slight, intercanine width and the pressure of the tongue, which occupies a smaller space [58]. Some methods to improve the stability of occlusion are the achievement of an ideal overjet and incisal stop, good intercuspation, and a durable retainer [59,60].

#### 3.4.3. Molar Extraction Patterns

In selected cases, extraction of the lower first [61], second [62], or third molars can be considered as an alternative camouflage option.

Extraction of first molars is generally indicated when these teeth are severely compromised or when posterior crowding and open-bite tendencies are present [61,63]. This procedure demands careful mechanics to prevent tipping of the second molar into the extraction space and to achieve proper root alignment. Second and third molars must be present to maintain posterior support, and maxillary second molars may require blocking to prevent overeruption [62]. A study showed that in the treatment of an open bite, the greater the posterior extraction, the greater the counterclockwise mandibular rotation will be [64].

Extraction of lower third molars can also facilitate distalization of the mandibular dentition or tipping of the second molars, thus aiding anteroposterior correction and vertical control [65,66]. When combined with skeletal anchorage, en-masse distalization of the mandibular arch can be achieved efficiently [67]. The extrusion of the upper third molars should be prevented by splinting them to the second molars [7]. Recent retrospective evidence suggests that third molar extraction, compared with premolar extraction, resulted in lower SNB angle and greater compensatory movement of the lower incisors and may lead to greater counterclockwise mandibular rotation and improved facial balance [27].

### 3.5. Non-Extraction Approaches

#### 3.5.1. Labial Fixed Appliance with Class III Elastics

This approach emphasizes the spontaneous dentoalveolar compensations typically observed in Class III malocclusions, i.e., the proclination of upper incisors and retroclination of lower incisors. These dental changes act as compensatory mechanisms for the underlying skeletal imbalance.

Appropriate patient selection is crucial for successful non-extraction treatment. Mild-to-moderate skeletal Class III relationships, acceptable facial profiles, and slight crowding in both arches are key factors that make non-surgical, non-extraction treatment feasible [68].

Intermaxillary Class III elastics are commonly used to achieve Class I molar and canine relationships; therefore, treatment success depends heavily on patient compliance [69]. Interproximal reduction of the enamel can also be considered in mild Class III cases [5].

Common side effects of intermaxillary Class III elastics include extrusion of upper molars, proclination of upper incisors, extrusion and retroclination of lower incisors, and distal tipping of lower molars [22]. Moreover, these effects may increase lower anterior facial height and induce a backward rotation of the mandibular plane [25].

To minimize such unwanted side effects, skeletal anchorage in combination with elastics can be employed [70].

Regarding soft tissue changes, recent retrospective evidence indicates a reduction in lower lip prominence, an increase in the nasolabial angle, and an increase in the Pog–NB distance following non-extraction treatment [26].

Since the sagittal forces generated by Class III elastics may influence the temporomandibular joint (TMJ), several studies have investigated their potential effects on joint morphology and function [71]. A recent study suggested that non-extraction Class III elastic therapy did not produce significant adverse effects on the condyle or articular disc [72].

In orthodontic camouflage of Class III malocclusion, differences among stainless steel, NiTi, superelastic NiTi, and copper NiTi archwires may influence treatment efficiency, as conventional NiTi and copper NiTi provide lower load–deflection rates, reduced force decay, and greater springback, allowing more continuous and controlled forces during incisor proclination [73]. On the contrary, stainless steel offers greater stiffness and superior torque expression, improving root control and anterior compensation during finishing [73]; superelastic NiTi may additionally enhance the delivery of more constant forces over a wider activation range, potentially improving the efficiency of dentoalveolar compensation [74].

#### 3.5.2. Self-Ligating Bracket Systems

Passive self-ligating systems are characterized by reduced friction between the bracket and the archwire, improved torque control, predictable uprighting of the lower teeth, and the potential for stable, significant dental expansion [24,75,76]. Expansion is achieved by increasing interpremolar and intermolar widths through the combined effect of reduced sliding resistance and the use of CuNiTi archwires [77,78].

Copper NiTi wires exhibit hysteresis, which creates consistent forces during deactivation over an extended period and promotes homogeneous load distribution along the archwire. Their lower elastic modulus compared with superelastic and conventional NiTi wires allows deformation under lower activation loads, which is associated with reduced patient discomfort and a lower risk of root resorption, particularly in the upper incisors [79]. Reduced friction also minimizes anchorage demands and helps achieve the required anterior torque [80].

Self-ligating systems have been reported to slightly reduce incisor proclination (approximately −1.5°) compared with conventional brackets, and may shorten chair time [81]. However, the literature presents conflicting evidence, as several systematic reviews have found no significant clinical advantages between conventional fixed appliances and passive self-ligating systems, except for a reduced archwire removal time in the latter [82,83].

In Class III camouflage cases, inverting the maxillary incisor bracket positions can help control upper incisor proclination. In the Damon self-ligating system, for example, the upper central and lateral incisor brackets have torque values of +15° and +6°, respectively; inverting these brackets allows for greater labial root torque, which is particularly beneficial in Class III patients [84].

The current evidence is limited to case reports, with no prospective studies evaluating the effects of camouflage treatment with self-ligating systems in adult Class III patients. Further research with larger samples and long-term follow-up is necessary to establish evidence-based recommendations.

#### 3.5.3. Lingual Fixed Appliances

Lingual appliances are predominantly used in adult patients because of their superior esthetics [85].

They have been reported to assist in open bite correction through myofunctional adaptation [86]. Moreover, the use of lingual brackets may induce postural changes in the tongue, shifting it upward and posteriorly [87,88].

Although lingual appliances exhibit a comparatively limited capacity for transverse expansion relative to conventional labial appliances, they offer advantages in vertical control and anterior torque management [89]. Consequently, they can be employed for open bite correction or for the camouflage treatment of skeletal Class III malocclusion with severe lower arch crowding [90]. In cases involving lower extractions, the use of a labial elastic chain can help control anterior torque and maintain the lower incisors upright, preventing unwanted retroclination [87].

It has also been reported that in cases involving the extraction of two lower premolars, lingual appliances provide effective torque control of the incisors, allowing for controlled retraction without excessive retroclination. These movements have been associated with remodeling of the dentoalveolar process [20,21].

Therefore, lingual fixed appliances may represent a valuable option for patients with high esthetic demands who require precise torque control following lower extractions. It has been hypothesized that these systems may also contribute to vertical dimension control. However, current evidence is limited to case reports, and no retrospective or prospective studies have yet evaluated these effects systematically.

#### 3.5.4. Clear Aligners

In recent years, clear aligner therapy has become increasingly popular, particularly among adult patients, due to its clinical efficacy, comfort, and minimal esthetic impact [91]. Clear aligners provide better periodontal health than fixed appliances, making them especially appealing to adults [92].

The use of clear aligners allows for lower arch distalization with acceptable predictability, particularly after third molar extraction, enabling the achievement of Class I molar relationships and improved anterior occlusion [93]. Lower distalization with clear aligners is a challenging movement, with a clinically achievable range of 2–3 mm [94]. Reported efficiencies are approximately 74% at the crown and 47% at the root level for the lower first molar, and around 74% for the crown of the second molar [95]. However, recent studies suggest that molar movement tends to occur primarily through distal tipping rather than true bodily distalization [34,35,36].

In patients presenting with anterior crossbite, the 1.5 mm occlusal disclusion produced by the aligner thickness facilitates anterior correction. Aligner therapy has also been reported to enhance sagittal anchorage control when combined with intermaxillary elastics [96] and to aid vertical control when unilateral elastics are used in asymmetrical Class III cases [97].

During distalization, clear aligners may help maintain proper lower incisor inclination and reduce distal tipping and molar extrusion [98], although the predominant pattern of movement remains coronal tipping rather than translation [35].

Clear aligners can be particularly useful in hyperdivergent Class III patients for maintaining vertical control during Class III elastic use [99]. Nevertheless, the posterior intrusive effect attributed to aligners may be limited, as masticatory forces are generally exerted when the aligner is not worn [100]. In hyperdivergent Class III cases with open bite, the use of temporary anchorage devices (TADs) in the lower arch can facilitate distalization while preventing mandibular clockwise rotation, without applying active intrusive forces on posterior teeth [101].

In adult patients with periodontal disease, clear aligner therapy represents a viable alternative due to its adaptability and the improved maintenance of oral hygiene. It has shown efficacy in managing alveolar bone defects, gingival recession, and tooth mobility [93], while minimizing risks of decalcification and periodontal deterioration associated with fixed appliances [92].

A recent prospective study [37] evaluating soft tissue changes in mild Class III cases reported that clear aligners, when combined with Class III elastics, slightly improved the facial profile without altering the lower third of the face, mainly by enhancing upper lip projection and promoting distalization of the lower arch [37].

Although current findings are promising, the evidence remains limited, and further prospective studies with larger samples and long-term follow-up are needed to validate the predictability and stability of Class III correction with clear aligner therapy.

In camouflage of Class III malocclusion with clear aligners, the viscoelastic behavior of thermoplastic materials, including polyethylene terephthalate glycol (PETG) and thermoplastic polyurethane (TPU), may significantly affect force delivery and treatment efficiency. Specifically, PETG generally exhibits greater initial stiffness and force delivery but may show more pronounced stress relaxation over time, whereas TPU may provide improved elastic recovery and sustained force application, potentially influencing the efficiency and predictability of sequential distalization [102]. In addition, optimized attachment geometries may enhance the transmission of moments and counteract tipping, thereby favoring bodily tooth movement during Class III camouflage [103].

#### 3.5.5. Carriere Motion III

One approach for non-surgical Class III correction involves the use of the Carriere Motion Class III (CM3) appliance [104,105]. Originally proposed for adult patients unwilling or unsuitable for orthognathic surgery, this method can produce significant occlusal improvement but generally yields limited facial profile enhancement, which remains primarily achievable through surgical correction. More recently, CM3 therapy has been proposed as a camouflage option for adolescent Class III patients [106].

The CM3 appliance, developed by Luis Carrière in 2015, consists of bilateral bars bonded to the mesial surfaces of the lower canines and first molars. Flat molar pads are centrally positioned on the clinical crowns, while hooks on the canine pads serve as attachment points for Class III elastics. The elastics are extended to buttons or tubes bonded to the most distal upper molars. Typically, ¼″ (8 oz) “Force 1” elastics are worn full-time for 4–5 months and replaced three times daily to minimize force degradation, which is more pronounced in latex-free elastics [104].

Anchorage is provided by the upper arch, commonly through a fixed appliance with rectangular wires or, alternatively, a thermoformed Essix retainer with buccal cutouts over the distal molars, where the buttons or tubes are bonded [104].

A shortened (“shorty”) version of the CM3 appliance may be used in selected cases—for example, between the lower first premolars and first molars when the canines are high, blocked out, or buccally displaced prior to treatment, or between the lower canines and second premolars in the absence of lower first molars [105].

Class III correction is achieved by establishing a so-called “Class I platform” [104]. The appliance induces distalization of the posterior mandibular segments as a unit, accompanied by a counterclockwise rotation of the posterior occlusal plane, resulting in an apparent mandibular repositioning. After achieving a Class I molar relationship, the lower canines are further distalized to create space for lower incisor retraction [105].

The appliance induces intrusion of the lower molars and extrusion of the lower canines, thereby altering the lower occlusal plane and contributing to the distal repositioning of the mandible [104]. Once overcorrection (typically an edge-to-edge molar relationship) is reached, treatment is completed with fixed appliances to align the dentition and finalize occlusion.

A retrospective study [106] reported minimal skeletal effects in the sagittal plane, with a slight increase in lower anterior facial height (approximately 2.5 mm). Improvement in molar relationship was associated with an anterior displacement of the upper molars relative to the maxilla (≈2 mm) and a posterior displacement of the lower molars relative to the mandible (≈2 mm). A net occlusal plane rotation of −3.1° was observed, suggesting that the correction achieved with CM3 is primarily dentoalveolar in nature [106].

#### 3.5.6. MEAW Technique

The Multiloop Edgewise Arch Wire (MEAW) technique is an advanced orthodontic approach developed in the 1960s and 1970s by Prof. Young H. Kim. Derived from the conventional Edgewise system established by Edward H. Angle, the MEAW technique incorporates multiple loops along the archwire to enhance three-dimensional control of tooth movement [107].

Initially designed to address complex malocclusions such as Class II, Class III, and anterior open bite cases, the MEAW technique employs light, continuous forces through individualized stainless-steel archwires (typically 0.016 × 0.022 in) with multiple loops between brackets. When combined with Class III intermaxillary elastics, these loops produce distal movement of the posterior mandibular teeth and allow differential tooth movement according to the patient’s needs [108].

Successful outcomes depend on careful patient selection, compliance, and biomechanical precision. Ideal candidates are patients with mild-to-moderate skeletal Class III malocclusion, acceptable facial profiles, and mild crowding in both arches [68,109]. The presence of mesially tipped lower posterior teeth on panoramic radiographs may further indicate suitability for MEAW therapy [68].

Correction of Class III malocclusion with the MEAW technique involves five main phases: alignment, elimination of occlusal interferences, establishment of a functional mandibular position, reconstruction of the occlusal plane, and achievement of a physiologic occlusion [107]. Following initial alignment with 0.014 in superelastic wires, interferences are eliminated using MEAW activation in both high- and low-angle Class III cases.

In the upper arch, anterior loops and uprighting bends promote labial tipping of the maxillary incisors to improve overjet. Controlled lingual root torque and retraction mechanics allow the lower incisors to be tipped lingually, further increasing overjet and correcting anterior crossbite. Reconstruction of the occlusal plane through uprighting of distally tipped molars and resolution of posterior crowding are characteristic effects of MEAW activation [109,110].

The use of Class III elastics may result in extrusion of upper molars and proclination of upper incisors, thereby increasing dentoalveolar compensation and potentially inducing clockwise rotation of the occlusal plane [68,111]. This effect can be detrimental in high-angle Class III cases. A retrospective study reported that in high-angle Class III patients, the MEAW technique primarily produces distal displacement of the lower posterior teeth rather than true posterior repositioning of the mandible or condyles [33].

To minimize undesirable effects, MEAW mechanics can be combined with maxillary skeletal anchorage and modified Class III elastics. This combination prevents labial tipping of the upper incisors and extrusion of the upper molars, while promoting distal tipping of mandibular molars and lingual inclination with extrusion of lower incisors [68,111]. The insertion of temporary anchorage devices (TADs) in the maxilla (typically between the second premolar and first molar) is technically easier, more comfortable, and less invasive than mandibular miniscrew placement [68,111,112]. Moreover, the use of Class III elastics from maxillary TADs can assist in correcting anterior crossbite [111].

The integration of TADs with MEAW mechanics is particularly indicated for high-angle Class III patients or those with an open bite tendency, offering improved vertical control and enhanced treatment stability.

#### 3.5.7. Skeletal Anchorage Methods

Among the various camouflage approaches for the treatment of adult Class III malocclusion, skeletal anchorage systems (such as temporary anchorage devices (TADs) and ramal plates) are primarily employed to achieve distalization of the lower arch. The use of skeletal anchorage has been associated with a reduced need for extraction therapy [113]. Lower molar distalization represents one of the most challenging orthodontic movements [114]. Several traditional methods have been proposed to achieve distal movement of the mandibular dentition, including mandibular headgear, lip bumpers, distal extension lingual arches, fixed appliances with Class III elastics, and edgewise loop mechanics [58]. However, these approaches often rely on the opposing arch for anchorage and can produce undesirable side effects such as tooth extrusion, clockwise rotation of the occlusal plane, and increased lower anterior facial height. In contrast, skeletal anchorage provides a stable, independent source of anchorage through the use of miniscrews or miniplates.

Mini-implants in the mandible can be placed in various sites, including the retromolar region or interradicular spaces [115].

Interradicular miniscrews allow only limited distalization (≤2–3 mm) due to the restricted interradicular space. They are preferred in mild discrepancies because of their minimally invasive placement and removal. For greater distalization along the occlusal plane, miniscrews can be positioned in the buccal shelf area, where high-density bone is present [32,116]. The insertion area is the mucogingival junction at the level of the lower second molar, 9 mm apical from the CEJ in hyperdivergent patients [117].

In cases with moderate skeletal or dental discrepancies, miniplates are preferred to avoid the risk of root contact. Ramal plates have been shown to produce more bodily distalization of the mandibular dentition and reduced distal tipping compared with miniscrews [118]. The resulting force vector may induce extrusion of the lower posterior teeth, which can be advantageous for compensating Class III relationships but must be managed carefully in hyperdivergent patients [119].

Miniplates exhibit greater mechanical stability and resistance to orthodontic forces than miniscrews due to the use of multiple fixation screws [120,121]. A systematic review reported failure rates of approximately 7.3% for miniplates compared with 16.4% for miniscrews [121].

A retrospective study found that miniscrews produced distal tipping and intrusion of lower molars, as well as bodily retraction of lower incisors and a reduction in mandibular plane angle, when compared with conventional Class III elastic therapy [30]. In contrast, ramal plates achieved greater distalization of the mandibular dentition (2.0–3.6 mm at the first molar) and induced a clockwise mandibular rotation [28,29].

The retromolar area is generally considered the preferred site for TAD placement in Class III cases due to adequate bone volume and minimal interference with adjacent roots, nerves, and blood vessels [122]. In cases of insufficient attached gingiva, the risk of miniscrew failure increases; in such cases, screws may be covered by the oral mucosa or alternatively placed in interradicular spaces [122].

Anatomical limitations must also be considered. The mylohyoid ridge and submandibular fossa define the posterior boundary for distalization. Contact between the distolingual root of the lower second molar and the mylohyoid ridge often limits distal movement to approximately 3 mm [123]. Therefore, cone beam computed tomography (CBCT) assessment is recommended before distalization to evaluate available space. Hypodivergent patients generally present greater posterior mandibular limits than hyperdivergent patients [116]. To minimize early root contact with the mylohyoid ridge, a rigid archwire with lingual root torque can be incorporated into the molars [116].

Due to the thicker cortical bone of the mandible, insertion torque and failure rates are generally higher than in the maxilla [124,125].

Care must also be taken to avoid soft tissue impingement in the retromolar area, as thick mucosa can limit distalization or contribute to relapse [43]. If the gingiva partially covers the lower second molar, inflammation may occur; in such cases, a minor gingivectomy or the application of light orthodontic forces is recommended [43].

Skeletal anchorage offers a reliable approach for mandibular distalization in Class III camouflage therapy, though its success depends on accurate site selection, anatomical assessment, and careful biomechanical control.

In skeletal anchorage–assisted camouflage of Class III malocclusion, miniscrew characteristics become particularly relevant due to the need for stable anchorage during anterior retraction and/or posterior dentoalveolar distalization, where uncontrolled tooth movement must be minimized to achieve dentoalveolar compensation without exacerbating skeletal discrepancies. Surface modifications such as acid-etching may enhance primary and secondary stability, which is critical in regions of limited cortical bone thickness frequently encountered in the anterior mandibular or posterior maxillary areas used for Class III mechanics [126,127]. Furthermore, optimal insertion torque is essential to balance primary stability and avoidance of bone microdamage, particularly in thin cortical bone sites [128]. Finally, failure rates are strongly influenced by bone density and loading protocol, with immediate or early loading protocols being clinically acceptable only when sufficient primary stability is achieved to sustain continuous Class III camouflage mechanics [129].

### 3.6. Smile Esthetics

In adult Class III patients undergoing camouflage treatment, careful attention should be given to smile esthetics. Class III malocclusion is often associated with increased exposure of the lower incisors, which may be further accentuated by the presence of an anterior crossbite. This tendency becomes more pronounced with age, as lower incisor exposure naturally increases over time [130]. Therefore, treatment objectives should include the prevention of further lower incisor extrusion and the promotion of upper incisor extrusion to enhance smile display. Several biomechanical strategies have been proposed to achieve these goals. The use of Class III elastics extending from the lower anterior region to upper posterior miniplates, or anterior box elastics combined with a reverse curve of Spee in the lower arch (or conversely in the upper arch with posterior elastics), has been reported to limit excessive lower incisor exposure [43]. Alternatively, placing the anterior brackets in a more gingival position or inverting the upper incisor brackets by 180° can provide additional crown torque and encourage upper incisor extrusion [131]. During alignment, particularly in adult patients or following extractions, the appearance of “black triangles” in the lower anterior region may compromise smile attractiveness [43]. Management options include interproximal enamel reduction, composite recontouring, or, in selected cases, periodontal regenerative surgery to restore interdental papilla height [132,133,134].

In general, positioning the upper anterior brackets more gingivally is a widely adopted technique to improve smile curvature in Class III patients, who often exhibit reduced incisor exposure and a flatter smile arc. This adjustment facilitates anterior tooth extrusion, increases upper incisor display, and assists in resolving anterior edge-to-edge or crossbite relationships [131,135]. Smile esthetics in orthodontic camouflage are not only determined by bracket positioning and torque control, but are also significantly influenced by the choice of appliance system. Labial fixed appliances generally allow more straightforward vertical positioning and torque expression; however, they may be associated with greater visibility of attachments and potential soft tissue profile effects during incisor retraction. Lingual appliances offer enhanced esthetic advantages and, due to their closer proximity to the lingual axis of resistance, may facilitate more effective torque control of the anterior dentition; nevertheless, they are technically more demanding in terms of vertical bracket placement and may present limitations in achieving precise incisor display control. Clear aligner systems, while highly favorable from an esthetic standpoint and particularly effective in managing mild-to-moderate incisor inclination changes, are constrained by reduced predictability in torque expression and vertical control, especially in cases requiring significant incisor intrusion or extrusion.

Overall, achieving balanced smile esthetics in Class III camouflage therapy requires coordinated control of vertical tooth position, bracket placement, and torque expression, while considering the patient’s age-related changes in dental exposure.

### 3.7. Clinical Recommendations and Stability

Excessive dental compensation in Class III camouflage treatment is prone to relapse, particularly when tooth movement exceeds the alveolar bone envelope. Lupi et al. [136] reported that iatrogenic effects such as root resorption and alveolar bone loss are more frequent in orthodontically treated adults, with approximately 37% of anterior sites showing more than 2 mm of alveolar bone loss. In Class III patients, alveolar dehiscence and gingival recession pose specific challenges, especially in the mandibular incisor region. Moreover, factors such as anterior open bite, tooth extraction, and excessive anterior retraction have been associated with increased risk of gingival recession [137,138].

Careful biomechanical control is essential to prevent root displacement outside the alveolar housing, which may result in dehiscence and soft tissue recession [139].

Excessive retroclination of the lower incisors beyond the biological limits has been identified as a risk factor for lingual bone dehiscence, potentially leading to gingival recession [140]. However, current evidence does not support the hypothesis that extraction-based camouflage treatment reduces the long-term risk of recession compared with non-extraction approaches [141,142].

According to a recent study [143], the sagittal relapse rate in Class III treatment in adults was about 30%. In this context, relapse is specifically defined as a reduction in overjet to less than 1 mm or the re-emergence of an improper occlusal relationship. Notably, the study emphasizes that extraction-based camouflage, frequently involving mandibular premolars, demonstrates superior long-term stability, whereas non-extraction protocols exhibit a statistically significant tendency toward relapse.

Relapse potential following orthodontic camouflage of Class III malocclusion may also vary according to the biomechanical approach employed. Multi-loop edgewise archwire (MEAW) therapy may provide effective dentoalveolar compensation and vertical control, but long-term stability may depend on neuromuscular adaptation and retention. The maintenance of the corrected occlusal relationships has been considered a key determinant of stability [33,144]. TAD-assisted distalization may offer greater anchorage control and more bodily tooth movement, potentially improving stability through reduced reliance on dental compensations alone, although relapse may still occur in cases of incomplete root control (molar distal tipping) or unresolved skeletal discrepancy [145]. Clear aligner–based camouflage may provide favorable short-term outcomes, but concerns remain regarding the long-term stability of complex movements such as distalization, torque control, and incisor compensation, given the limitations in movement predictability and retention dependence [91,94].

Following completion of camouflage therapy, an appropriate retention protocol is crucial to prevent space reopening and relapse due to tongue pressure or lower incisor proclination [58,146]. Long-term stability following orthodontic camouflage of Class III malocclusion depends not only on treatment mechanics but also on individualized retention strategies extending beyond conventional short-term protocols. A bonded 3 × 3 retainer combined with a removable wraparound retainer is typically recommended full-time for one year, followed by nighttime wear for an additional year [147].

Although full-time removable retention during the first post-treatment year is commonly prescribed, prolonged retention may be advisable in camouflage cases because dentoalveolar compensations, particularly incisor inclination changes, may be prone to relapse over time [148,149]. In this context, retention protocols should be tailored according to the type and magnitude of compensation achieved, with combined fixed and removable retention often advocated to enhance long-term stability. Fixed lingual retainers may be particularly beneficial for maintaining mandibular incisor position, whereas removable retainers may contribute to preserving transverse relationships and occlusal settling [150,151]. Furthermore, reduced nighttime wear rather than complete discontinuation has been proposed in high-relapse-risk cases, especially when camouflage relies on substantial anterior compensation or distalization mechanics [149]. Therefore, retention in Class III camouflage should be viewed as a long-term stability strategy rather than a finite post-treatment phase.

The long-term success of orthodontic camouflage in Class III malocclusion may depend not only on dentoalveolar compensation and skeletal anchorage mechanics, but also on functional factors such as tongue posture and orofacial muscular balance [152]. Aberrant tongue posture, low tongue resting position, and dysfunctional swallowing patterns may contribute to unfavorable dentoalveolar forces capable of influencing incisor inclination, transverse stability, and post-treatment relapse, particularly in cases relying on significant anterior compensation [147]. In this context, orofacial myofunctional therapy has been proposed as an adjunctive approach aimed at improving tongue posture, lip competence, and neuromuscular equilibrium, potentially supporting the stability of orthodontic camouflage outcomes [153]. Although evidence regarding its isolated effect on tooth movement remains limited, myofunctional intervention may be particularly relevant in patients susceptible to relapse, where correction of functional etiologic factors could complement mechanical retention strategies [42,147,154].

Continuous follow-up is necessary to monitor periodontal health and ensure long-term stability, as relapse and mucogingival changes remain key concerns in adult Class III camouflage treatment.

### 3.8. Limitations of Camouflage

Despite its effectiveness in selected cases, orthodontic camouflage presents important biomechanical and esthetic limitations. From a biomechanical perspective, treatment relies on dentoalveolar compensation to mask the underlying sagittal discrepancy, often combined with limited mandibular distalization, since skeletal growth is complete and orthopedic correction is no longer feasible in adults [44]. These compensatory movements must remain within the anatomical boundaries of the alveolar bone, as excessive incisor proclination or retroclination may increase the risk of alveolar dehiscence, fenestrations, periodontal compromise, and reduced long-term stability [41,155].

In particular, excessive distal tipping of the lower incisors has been associated with relapse, emphasizing the importance of maintaining proper incisor inclination [42].

In addition, camouflage may have limited ability to fully correct associated vertical or transverse discrepancies, potentially resulting in incomplete occlusal correction.

From an esthetic perspective, mandibular prognathism or maxillary retrusion may persist, restricting facial profile improvement compared with orthognathic surgery. For this reason, camouflage is indicated in mild-to-moderate Class III malocclusions, where acceptable facial esthetics can be maintained or modestly improved [4,14]. In borderline cases, careful diagnosis is critical, as cephalometric thresholds such as an ANB angle ≤ −4° or an incisor–mandibular plane angle ≤ 83° have been associated with better outcomes following surgical correction [156,157]. Additional parameters, including the Wits appraisal and Holdaway angle, also play an important role in distinguishing patients suitable for camouflage from those who may benefit more from surgery [4,39].

### 3.9. Limitations and Strengths of the Review

As a narrative review, this study presents inherent methodological limitations. A comprehensive database search without restrictions on year, language, or publication status was performed to minimize selection bias. Inclusion and exclusion criteria were explicitly defined to ensure reproducibility; however, the included studies were not formally assessed for methodological quality or risk of bias, which may limit the strength of the conclusions. The present review should be interpreted as a qualitative synthesis, and its conclusions are limited by the non-systematic search strategy and the absence of a meta-analysis.

The current evidence on camouflage treatment in adult Class III patients remains not high. Most available data derive from retrospective observational studies, with the most recent studies on TAD-assisted distalization and many isolated case reports. Further prospective, ideally multicenter and randomized, studies are needed to compare the effectiveness and long-term stability of the various therapeutic protocols.

Despite these limitations, the narrative approach allows for broader conceptual integration of findings, encouraging theoretical reflection and the generation of new research hypotheses. Given the scarcity of high-level evidence, this review provides a realistic synthesis of current knowledge on camouflage strategies for adult Class III malocclusion.

## 4. Conclusions

Orthodontic camouflage appears to be an effective treatment for adult patients with mild-to-moderate Class III malocclusion when dentoalveolar compensations can be achieved within the biological boundaries of tolerability. The current review indicates that alternative techniques may be effective depending on the clinical presentation. However, the available scientific evidence on this topic remains limited and is predominantly of low quality. Fixed appliances in combination with Class III elastics can be most advantageous in patients with slight skeletal disharmony, acceptable soft tissue profiles, and high compliance, while evidence suggests that clear aligners and lingual appliances may be necessary for adults with high esthetic demands or who require greater torque and vertical control. Extraction-based techniques, especially lower premolar extraction, seem to provide efficient correction in patients with either lower incisor proclination and crowding, while single-incisor extraction is reserved for selected patients with an anterior tooth size ratio discrepancy and with stable posterior intercuspation. Skeletal anchorage systems, including mandibular buccal shelf miniscrews and ramal plates, appear to be the most appropriate and effective for patients who require significant sagittal corrections or bodily distalization. However, the evidence should be enhanced by high-quality studies. The strength of camouflage in all procedures is expected to be primarily determined by four diagnostic parameters: (1) the extent of skeletal discrepancy to the patient’s dentoalveolar compensatory capacity, (2) the morphology of the mandibular symphysis and the limits of upper and lower incisor inclination, (3) the vertical pattern of the patient and (4) the changes in the soft tissue profile in response to treatment. Camouflage can take on a risky (and oftentimes unstable) appearance when planned moves go beyond the alveolar housing, when lower incisors are already retroclined, or when the facial profile is markedly concave and probably is unlikely to benefit from dental compensation alone. Finally, patient selection is the key determinant. An evaluation of cephalometric factors, periodontal and anatomical limits, esthetic requirements, and adverse side effects may be reviewed before choosing camouflage as an alternative to orthognathic treatment. When appropriately indicated and biomechanically controlled, camouflage therapy can result in meaningful functional and esthetic benefits; however, clinicians must acknowledge its limitations and the need for additional high-quality evidence to clarify the most predictable and stable strategies for adult Class III treatment.

## Figures and Tables

**Table 1:** Summary of observational studies for camouflage of Class III malocclusion in adults.

Study	Sample	Treatment Modality	Key Findings
Downarowicz et al. 2009 [22]	4 lower extraction/6 non-extraction/10 facemask (14–39 y)	Lower extraction vs. non-extraction camouflage	Similar skeletal changes; dentoalveolar compensation observed
Burns et al. 2010 [23]	30 treated/30 untreated controls (12.4 ± 1 y)	Fixed appliances + Class III elastics vs. controls	Sagittal improvement (Wits appraisal) and profile enhancement in treated group
Cheng et al. 2020 [24]	30 mild or moderate with MBT technique/19 mild or moderate with transmission technique/19 severe with transmission/19 severe with MBT (18–21 y)	Fixed appliances + Class III elastics	Improved profile; reduced incisor compensation with transmission system
Wu et al. 2022 [25]	27 (>18 y)	Fixed appliances + Class III elastics	Increased lower facial height; mandibular clockwise rotation
Li et al. 2024 [26]	80 (mean 18.7 y)	Fixed appliance	Soft tissue profile improvement
Thiem et al. 2024 [20]	25 (mean 26.8 y)	Lingual camouflage with extractions (lower premolars)	Effective retraction without excessive incisor retroclination
Wiechmann et al. 2025 [21]	25 (mean 26.8 y)	Lingual camouflage with extractions (lower premolars)	Stable dentoalveolar adaptation during retraction (follow-up 3.1 years)
Liu et al. 2025 [27]	14 with lower extractions/17 with third molars extractions and TADs (mean 19.5 y)	Lower premolar extractions vs. third molar extractions and TAD-assisted camouflage	Different mechanics, similar soft tissue outcomes
Yu et al. 2016 [28]	22 (mean 23.9 y)	Ramal plate distalization	Effective mandibular distalization with limited extrusion and rotation of the molars
Yeon et al. 2022 [29]	20 with miniscrews/20 with ramal plates (mean 25.5 y)	Miniscrews vs. ramal plates	More molar intrusion and mandibular counterclockwise rotation with miniscrews More distalization and mandibular clockwise rotation with ramal plate
Nakamura et al. 2017 [30]	11 with TADs/12 with Class III elastics (18–25 y)	TAD distalization vs. elastics	TADs favored in high-angle cases
Azeem et al. 2018 [31]	30 with lower premolar extractions/30 with elastics or TADs (18 y)	Extractions vs. distalization	Comparable efficiency and occlusal outcomes
Ye et al. 2013 [32]	10 with miniscrews in the retromolar area/9 with miniscrews in the posterior area	Retromolar vs. posterior miniscrews	Retromolar anchorage improved bodily distalization
Guo et al. 2020 [33]	20 (mean 22.3 y)	MEAW	Correction mainly through dentoalveolar compensation
Wu et al. 2021 [34]	20 (>18 y)	Clear aligner distalization	Distalization; mainly tipping movement
Rota et al. 2022 [35]	21 (mean 25.6 y)	Clear aligners + Class III elastics	Effective molar correction with tipping tendency
Han et al. 2021 [36]	32 (mean 24.8 y)	Clear aligners + elastics vs. TADs	Overcorrection may be needed; tipping is prevalent
Lione et al. 2025 [37]	19 (mean 26 y)	Clear aligners + Class III elastics	Favorable upper lip changes, no change in the facial lower third

**Table 2:** Qualitative appraisal of the available evidence for each treatment modality.

Technique	Strength of Evidence	Study Design	Qualitative Comment
Extraction-based approaches	Moderate	Historical and observational studies	Moderate evidence, predictable outcomes; predominantly observational data.
Non-extraction	Low-to-moderate	Case reports and retrospective analyses	Low-to-moderate evidence, context-dependent effectiveness (mild skeletal discrepancies with good patient cooperation); limited long-term data.
Self-ligating systems	Low	Case reports and narrative descriptions	Low evidence, no consistent advantage over conventional systems.
Lingual appliances	Low	Case reports and small case series	Low evidence, consistent esthetic and biomechanical outcomes (torque control).
Clear aligners	Low	Case reports and small prospective studies	Low evidence, heterogeneous outcomes in mild cases.
Carriere Motion III	Low-to-moderate	Retrospective studies and case reports	Low-to-moderate evidence, consistent dentoalveolar effects; limited skeletal impact.
MEAW technique	Low	Case reports	Low evidence, consistent vertical control; based on descriptive studies.
Skeletal anchorage	Moderate	Retrospective studies and case series	Moderate evidence, consistent biomechanical effects (mandibular distalization); lack of randomized trials.

**Table 3:** Selection criteria of patients for camouflage treatment: characteristics that determine whether camouflage treatment is feasible.

Characteristic	
Sagittal discrepancy *	Not severe (mild-to-moderate; possible including cephalometric cut-off values for decision making)
Upper incisors	Favorably inclined (retroclined)
Lower incisors	Favorably inclined (proclined)
Alveolar bone support	Mandibular symphysis should be sufficiently thick, which allows the lower incisors to be moved back
Vertical dimension and growth pattern	Not excessive hyperdivergence
Facial aesthetics Patient preferences, compliance, and limitations	Mild concavity Treatment acceptable for the patient

* this aspect must take precedence over the next ones.

**Table 4:** Summary of characteristics of camouflage techniques for adult Class III malocclusion.

**A. Extraction-based camouflage approaches**						
**Technique**	**Clinical Indications**	**Advantages**	**Limitations/Risks**	**Expected Tooth Movements**	**Level of Evidence**	**Ideal** **Patient** **Profile**
Upper second premolar and lower first premolar extraction	Moderate skeletal Class III with dental compensation; negative overjet; moderate/severe crowding; acceptable or slightly concave profile	Favors correction of anterior crossbite while preserving maxillary incisor position; allows significant retraction of lower incisors; improves sagittal dental relationship	Risk of excessive lower incisor retroclination; increased facial concavity if soft tissues are unfavorable; complex space management	Significant lower incisor retraction; mesialization of maxillary posterior segment; improvement of overjet and canine relationship	Case reports and retrospective studies	Adults with moderate Class III discrepancy, proclined lower incisors, adequate maxillary incisor position
Lower premolar extraction (first or second)	Lower arch crowding; proclined lower incisors; moderate skeletal discrepancy; need to maintain upper incisor support	Facilitates correction of negative overjet; allows retraction of lower incisors	Potential increase in facial concavity; extraction space may be insufficient for large retraction	Lower incisor retroclination; improved molar/canine relationship	Mainly case reports; limited retrospective evidence	Patients with proclined lower incisors and protruded lower lip
Single lower incisor extraction	Mild anterior crossbite; favorable Bolton ratio; good posterior occlusion	Reduced treatment time; minimal change to arch form	Risk of black triangles; tooth size discrepancies; overjet increase	Rapid alignment; minimal arch width alteration	Systematic reviews + case reports	Patients with mild discrepancy, stable posterior occlusion, and minimal esthetic impact
Lower molar extraction (first, second, or third molars)	Severely compromised molars; open bite tendency; need for posterior uprighting or rotation	Facilitates distalization; may improve vertical control	Complex mechanics; risk of tipping of second molars	Distalization up to ~3 mm, especially with skeletal anchorage	Case reports	Patients with compromised molars or requiring posterior uprighting/vertical improvement
**B. Non-extraction approaches**						
**Technique**	**Clinical** **Indications**	**Advantages**	**Limitations/Risks**	**Expected Tooth Movements**	**Level of Evidence**	**Ideal Patient Profile**
Class III elastics with labial fixed appliances	Mild–moderate Class III discrepancy; acceptable facial profile; patient compliance	Simple mechanics; non-invasive; widely available	Extrusion of upper molars; retroclination of lower incisors; clockwise mandibular rotation; vertical increase	Upper incisor proclination; lower incisor retroclination; improvement of 1–2 mm in molar relationship	Mainly case reports; limited retrospective studies	Normo- or hypodivergent pattern; mild crowding; lower incisors not excessively retroclined
Passive self-ligating systems	Mild–moderate Class III requiring dental expansion and torque control	Reduced friction; potentially improved torque expression; lower anchorage demands	Conflicting evidence on effectiveness; limited expansion capacity	Slight reduction in incisor proclination; transverse dental expansion not significant	Case reports; no observational studies	Patients requiring modest arch development and controlled incisor torque
Lingual appliances	High esthetic demand; need for strong torque control	Excellent torque control; improved esthetics; suitable for adults	Limited transverse expansion; technical complexity	Effective control of incisor inclination; controlled retraction in extraction cases	Case reports	Adult patients requesting esthetic treatment
Clear aligners	Mild–moderate Class III; esthetic preference; need for vertical control	Comfort, hygiene, improved vertical control; better anchorage with elastics	Distalization mainly via tipping; limited bodily movement; compliance-dependent	Mandibular distalization of 2–3 mm; acceptable incisor torque control	Retrospective studies + case reports	Adults with mild–moderate discrepancy, good periodontal status, and high esthetic expectations
Carriere Motion III	Dental Class III; need for early sagittal correction	Rapid initial molar correction; simple patient workflow	Predominantly dentoalveolar effects; limited skeletal improvement	Mandibular distalization; posterior occlusal plane rotation (~3°)	Retrospective clinical studies	Adults with dental Class III, mild–moderate sagittal discrepancy, and good compliance
MEAW (Multiloop Edgewise Archwire) with Class III elastics	Complex Class III cases; altered occlusal plane; open bite tendency	Excellent 3D control; ability to reshape occlusal plane; effective dental decompensation	High clinician skill required; risk of undesired vertical effects; high patient compliance	Uprighting of molars; increased overjet; reconstruction of the occlusal plane	Case reports + a few retrospective studies	Patients with moderate discrepancy and need for vertical and sagittal control
**C. Camouflage techniques using skeletal anchorage**						
**Technique**	**Clinical Indications**	**Advantages**	**Limitations/Risks**	**Expected Tooth Movements**	**Level of Evidence**	**Ideal Patient Profile**
Interradicular miniscrews (mandibular)	Mild–moderate Class III requiring about 2 mm distalization	Minimally invasive; easy placement and removal	Limited space; risk of root proximity; limited distalization	Distalization ~2 mm; improved incisor torque control	Retrospective studies	Patients with mild discrepancy, adequate interradicular space, and healthy periodontium
Buccal shelf miniscrews	Need for moderate distalization (>2 mm)	High anchorage capacity; reduced root interference	Soft-tissue thickness; patient discomfort	Distalization up to 2–3 mm; improved bodily movement compared with elastics	Retrospective studies	Hypodivergent patients with adequate anatomical space
Miniplates/Ramal plates	Moderate–severe discrepancy in non-surgical patients	Strongest anchorage; promotes bodily distalization	Requires minor surgery; higher cost	Distalization 2–4 mm; minimal molar tipping; possible clockwise mandibular rotation	Retrospective studies	Non-surgical adults with larger anteroposterior discrepancy

## Data Availability

No new data were created or analyzed in this study.
